# Data-Driven multi-Contrast spectral microstructure imaging with InSpect: INtegrated SPECTral component estimation and mapping

**DOI:** 10.1016/j.media.2021.102045

**Published:** 2021-07

**Authors:** Paddy J. Slator, Jana Hutter, Razvan V. Marinescu, Marco Palombo, Laurence H. Jackson, Alison Ho, Lucy C. Chappell, Mary Rutherford, Joseph V. Hajnal, Daniel C. Alexander

**Affiliations:** a Centre for Medical Image Computing, Department of Computer Science, University College London, UK; b Centre for the Developing Brain, Kings College London, London, UK; c Biomedical Engineering Department, Kings College London, London, UK; d Women’s Health Department, King’s College London, London, UK

**Keywords:** MRI, Microstructure imaging, Diffusion-relaxation MRI, Inverse Laplace transform, Unsupervised learning, Quantitative MRI, Placenta MRI

## Abstract

We introduce and demonstrate an unsupervised machine learning technique for spectroscopic analysis of quantitative MRI experiments. Our algorithm supports estimation of one-dimensional spectra from single-contrast data, and multidimensional correlation spectra from simultaneous multi-contrast data. These spectrum-based approaches allow model-free investigation of tissue properties, but require regularised inversion of a Laplace transform or Fredholm integral, which is an ill-posed calculation. Here we present a method that addresses this limitation in a data-driven way. The algorithm simultaneously estimates a canonical basis of spectral components and voxelwise maps of their weightings, thereby pooling information across whole images to regularise the ill-posed problem. We show in simulations that our algorithm substantially outperforms current voxelwise spectral approaches. We demonstrate the method on multi-contrast diffusion-relaxometry placental MRI scans, revealing anatomically-relevant sub-structures, and identifying dysfunctional placentas. Our algorithm vastly reduces the data required to reliably estimate spectra, opening up the possibility of quantitative MRI spectroscopy in a wide range of new applications. Our InSpect code is available at github.com/paddyslator/inspect.

## Highlights

• Unsupervised learning technique for spectroscopic analysis of quantitative MRI.
• Shares information across voxels to improve estimation of multi-dimensional or single-dimensional spectra.
• Spectral maps are dramatically improved compared to existing approaches.
• Can potentially identify and map tissue environments; in placental diffusion-relaxometry MRI we demonstrate that it identifies components that correspond to distinct tissue types.

## Graphical abstract

**Figure:**
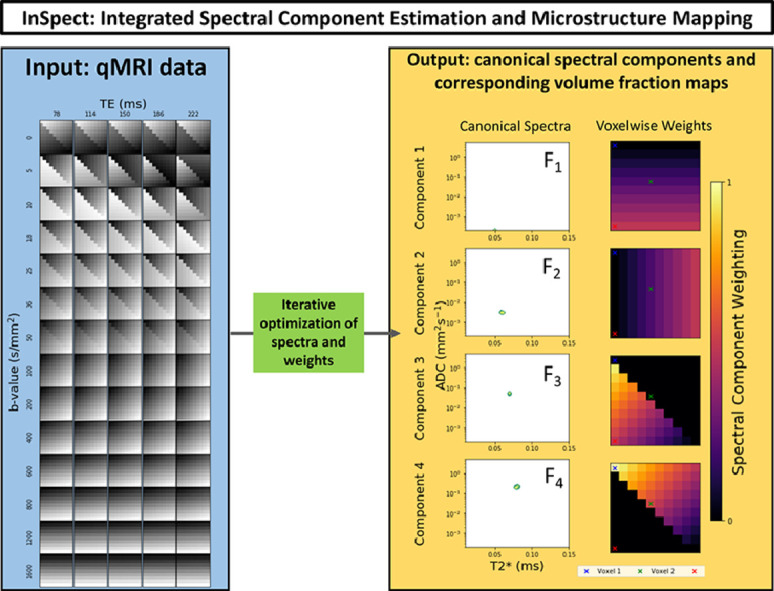

## 1 Introduction

Quantitative MRI can measure and map physical and chemical quantities that strongly relate to underlying tissue structure and function. A convenient, and data-driven, way to analyse quantitative MRI data is to assume a population of spins with a distribution of quantities (e.g. relaxivity, diffusivity) that are encoded in a one-dimensional, or multidimensional correlation, spectrum. By estimating such distributions, multiple microstructural components can be distinguished without making a-priori modelling assumptions, such as fixing the number of tissue compartments. This approach, which we refer to as quantitative MRI spectroscopy in this paper, has the potential to provide novel biomarkers (Bai et al., 2014). Quantitative MRI spectroscopy has been demonstrated for single-contrast approaches, including T2 component analysis of multi-echo relaxometry data, as has been used to image myelin (Alonso-Ortiz, Levesque, Pike, 2015, Nagtegaal, Koken, Amthor, Bresser, Vos, Doneva, 2020) and luminal water (Sabouri, Chang, Savdie, Zhang, Jones, Goldenberg, Black, Kozlowski, 2017, Devine, Giganti, Johnston, Sidhu, Panagiotaki, Punwani, Alexander, Atkinson, 2019).

Quantitative MRI spectroscopy is also an attractive technique for analysing MRI experiments that concurrently measure multiple MR properties such as T1, T2, and diffusivity. By providing information on correlations and couplings between complementary MR properties, this approach can resolve distinct microstructural compartments that are indistinguishable with a single contrast. Various types of multi-contrast correlation spectroscopy have been demonstrated, such as relaxometry-relaxometry (English et al., 1991) and diffusion-relaxometry (Van Dusschoten et al., 1996). Several papers have leveraged recent advances in scanner hardware to extend these ideas into imaging, in the T1-diffusion (De Santis, Barazany, Jones, Assaf, 2016, De Santis, Assaf, Jeurissen, Jones, Roebroeck, 2016), T2-diffusion (Veraart, Novikov, Fieremans, 2018, Kim, Doyle, Wisnowski, Kim, Haldar, 2017, Melbourne, Aughwane, Sokolska, Owen, Kendall, Flouri, Bainbridge, Atkinson, Deprest, Vercauteren, David, Ourselin, De Almeida Martins, Topgaard, 2018, Lampinen, Szczepankiewicz, Mårtensson, van Westen, Hansson, Westin, Nilsson, 2020, De Almeida Martins, Tax, Reymbaut, Szczepankiewicz, Jones, Topgaard, 2020, Reymbaut, Martins, Tax, Szczepankiewicz, Jones, Topgaard, Gong, Tong, He, Sun, Zhong, Zhang, 2020), T1-T2-diffusion (Benjamini and Basser, 2017), T2*-diffusion (Slator et al., 2019b), and T1-T2*-diffusion (Hutter et al., 2018) domains.

**Fig. 1:**
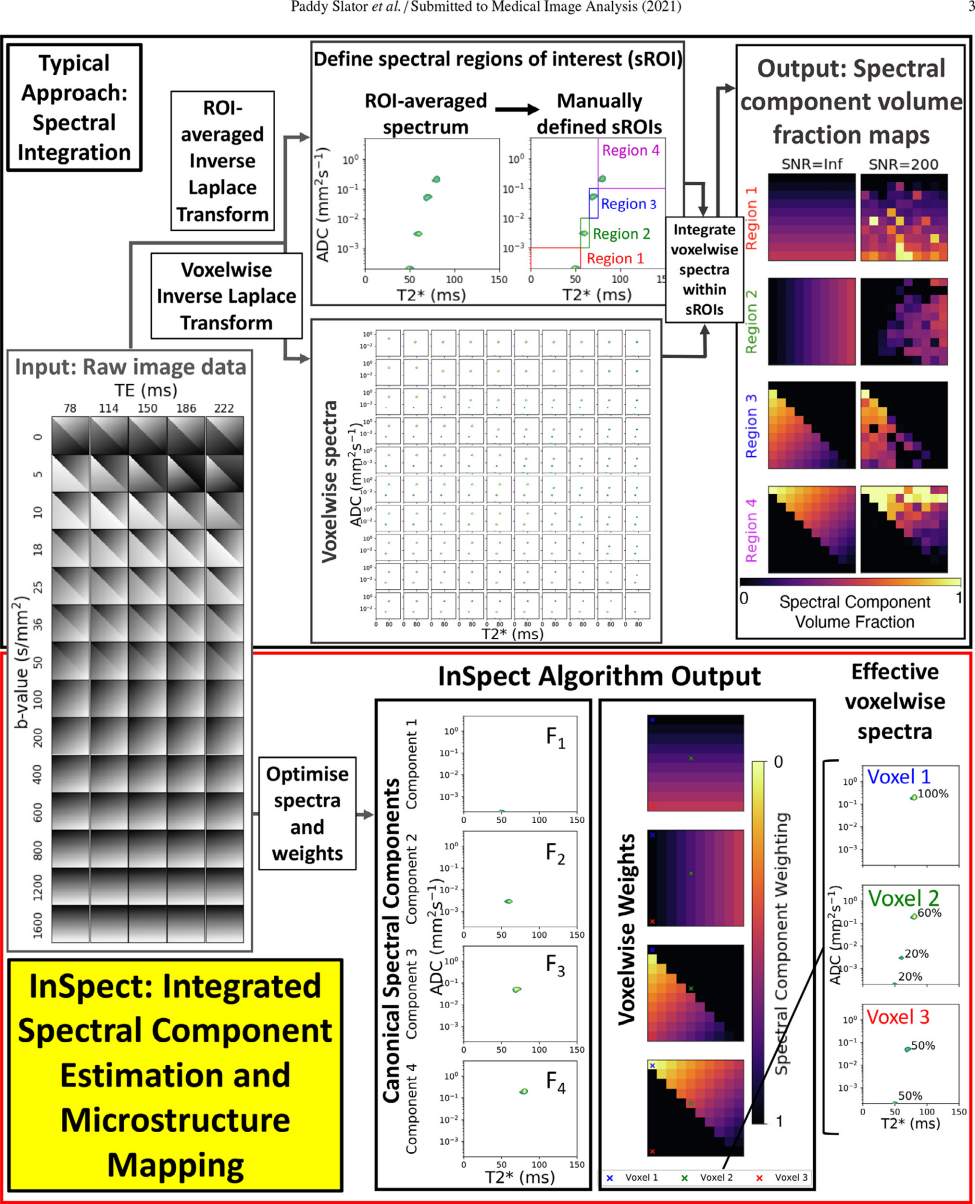
Schematic comparison of voxelwise integration and InSpect for spectral estimation from combined T2*-diffusion data. Top panel: derivation of volume fraction maps from multi-contrast MRI using spectral integration. In spectral integration, spectral regions of interest (sROI) are manually identified, typically by examining spectra calculated from the signal averaged across image ROIs. Spectra are also calculated for every image voxel. Volume fraction maps are thus calculated by integrating the voxelwise spectra in the sROIs. Bottom panel: Illustration of InSpect applied to multi-contrast MRI. The algorithm simultaneously infers a set of M canonical spectral components (e.g. $F_{1}$ to $F_{4},$ these examples here have single modes but they can equally be multimodal) and their corresponding voxelwise weightings.

A general continuum model for quantitative MRI spectroscopy gives a Fredholm integral equation on the MR signal (Benjamini and Basser, 2020), or a Laplace transform in the specific exponential decay case, e.g. for diffusivity or relaxivity. The spectrum can be estimated using regularised inversion of this integral (English et al., 1991), although this is highly ill-posed. Estimating spectra in each image voxel independently therefore requires unrealistically high signal-to-noise ratio (SNR). Moreover, to derive meaningful quantitative maps from voxelwise spectra typically involves a procedure known as *spectral integration*; see top panel Fig. 1 for a two-dimensional example. In spectral integration, the user first manually identifies regions of the spectrum (termed spectral regions of interest, sROI). These sROIs are typically found by identifying prominent features in spectra estimated from the signal averaged over image regions of interest (ROIs). Scalar indices are then calculated by numerical integration of each voxel’s reconstructed spectrum over these sROIs (Mackay, Whittall, Adler, Li, Paty, Graeb, 1994, Kim, Doyle, Wisnowski, Kim, Haldar, 2017, Benjamini, Basser, 2017).

Recently, methods have been proposed for increasing the robustness of voxelwise spectral fits, utilising marginal distributions (Benjamini and Basser, 2016) or spatial regularisation (Kim et al., 2017a). These methods can improve inversion stability and thus give more meaningful quantitative maps. However, inherent limitations remain. In particular, they rely on ad-hoc choices of regularisation terms and manually defined sROIs. A recent technique automatically identifies these spectral integration regions (Pas et al., 2020), but restricts to rectangular and non-overlapping sROIs, depends on a user-defined threshold value, and still requires voxelwise spectral estimation.

Here we present a method which addresses these limitations in a data-driven way. It simultaneously estimates a canonical basis of spectral components for a whole image (or a data set comprising multiple images), and the voxelwise weighting factors of each component. We build on previous work that exploits data redundancies in nuclear magnetic resonance (NMR) datasets, including (a) COmponent REsolved (CORE) NMR (Stilbs et al., 1996) - a technique for analysing Fourier Transform Pulsed-Gradient Spin-Echo (FT-PGSE) data sets that has also been applied to NMR images (Stilbs, 1998) - and (b) our previously published discrete InSpect algorithm (Slator et al., 2019a). We hence derive a technique that estimates a set of underlying spectra and corresponding maps of their relative contributions in each voxel. This allows us to capture smooth changes in assignment to spectral components across the image, rather than forcing hard categorisation of pixels into a small set of components, which discards subtle variation. Our approach is inspired by the fact that the MR signal comes from a multitude of microstructural environments, motivating the estimation of a set of canonical bases disentangling these environments. Moreover, the spectra and maps outputted by our algorithm are inherently interpretable, unlike many AI - particularly deep learning - techniques (Castelvecchi, 2016, Lundervold, Lundervold).

Unlike standard inversion approaches, the InSpect algorithm introduced here exploits the large amount of information shared among voxels, dramatically reducing the SNR required for stable inversion, hence enabling quantitative MRI spectroscopy in a wide variety of new situations. The method provides a natural lower-dimensional representation enabling standard downstream analysis of images without manual division of the spectral domain (into sROIs) or image segmentation (into image ROIs). Fig. 1 visualises the existing voxelwise method (top panel) and our proposed InSpect algorithm (bottom panel) side-by-side. In this paper we demonstrate InSpect by estimating multidimensional correlation spectra from combined T2*-diffusion data, but we emphasise that our algorithm is applicable to data of any dimension.

The paper proceeds as follows: we first describe the assumptions underlying the continuous InSpect algorithm, then derive the iterative optimisation algorithm. We demonstrate InSpect first in simulations, then on in-vivo diffusion-relaxometry placental MRI data. Our Matlab (The MathWorks, Natick, MA) implementation of InSpect is available at github.com/paddyslator/inspect.

## 2 Methods

### 2.1 Related methods

InSpect is based on a continuum model, which assumes that single voxels contain spins with a spectrum of MR properties. For a general n-dimensional multi-contrast MRI experiment the voxel signal is

(1) $$S\left(t\right)=\int \cdots \int F\left(\omega \right)K\left(t,\omega \right)\;d\omega_{1}\cdots d\omega_{n}$$

where t is a vector of experimental parameters which are varied to yield contrast in intrinsic MR properties ω, via the specific form of the kernel K(t,ω). F is the n-dimensional spectrum over ω, i.e. the distribution of these values across all spins. For example, in two-dimensional T2*-diffusivity (or T2-diffusivity) imaging, $t=(b,T_{E}),$ the b-value and echo time (TE); $\omega =(ADC,T_{2}^{*}),$ the apparent diffusion coefficient and T2*; $K\left(t,\omega \right)=\exp\left(-T_{E}/T_{2}^{*}\right)\exp\left(-bADC\right)$ is the kernel; and F is the T2*-ADC spectrum.

The standard approach for estimating the spectrum from a general n- dimensional quantitative MRI experiment, following (Menon, Allen, 1991, English, Whittall, Joy, Henkelman, 1991, Ronen, Moeller, Ugurbil, Kim, 2006), proceeds as follows. Equation (1) is first discretised onto an n-dimensional grid, with lengths defined by the user-defined vector $N_{\omega }=\left(N_{\omega_{1}},\ldots ,N_{\omega_{n}}\right)$. This gives the following signal expression

(2) $$S\left(t\right)=\sum_{l_{1}=1}^{N_{\omega_{1}}}\cdots \sum_{l_{n}=1}^{N_{\omega_{n}}}F\left(\omega (l_{1},\ldots ,l_{n})\right)K\left(t,\omega (l_{1},\ldots ,l_{n})\right).$$

By choosing an ordering of the elements of the n-dimensional grid coordinates, the signal for all MR encodings in the experiment can thus be written in matrix form

(3) S=KF

where S is a column vector, length $N_{s},$ of the signals at each MR encoding, K is an $N_{s}$ by $\prod_{l=1}^{n}N_{\omega_{l}}$ matrix of discretised kernel values, and F is an $\prod_{l=1}^{n}N_{\omega_{l}}$ length column vector of spectrum values. The spectrum F is then calculated with regularised non-negative least squares

(4) $$F=\underset{F\geq 0}{arg\;min}{\;∥KF-S∥}_{2}^{2}+\alpha {∥F∥}_{2}^{2}.$$

By solving Equation (4) in each voxel, the spectrum can be estimated across a whole image. Volume fraction maps are then produced by numerically integrating voxelwise spectra over user-defined sROIs, (Kim, Wisnowski, Haldar, 2017, Benjamini, Basser, 2017, Mackay, Whittall, Adler, Li, Paty, Graeb, 1994), see top panel Fig. 1. However low SNR can lead to noisy spectrum estimates and hence poor spatial maps.

CORE NMR is an existing method that produces spatial maps by sharing information across voxels and hence increases the effective SNR. CORE assumes a fixed number of components across an image (Stilbs, 1998) (or equivalently an NMR spectra (Stilbs et al., 1996)). In particular, for the two-dimensional combined T2-D imaging application (Stilbs, 1998), assuming M components, the signal for a single MR encoding in voxel n is

(5) $$S_{n}\left(b,T_{E}\right)=\sum_{m=1}^{M}z_{nm}\exp\left(-T_{E}/T_{2m}\right)\exp\left(-bADC_{m}\right)$$

where $z_{nm}$ is the weighting of component m in voxel n, and $T_{2m},$ $ADC_{m}$ are the single-valued MR properties (i.e. not spectra) for each component. Similarly to Equation (3) the signal for all MR encodings in voxel n can be written in vector form as

(6) $$S_{n}=\sum_{m=1}^{M}z_{nm}\exp\left(-T_{E}/T_{2m}\right)\exp\left(-bADC_{m}\right)$$

where $T_{E}$ and b are column vectors, length $N_{s},$ of the MR encoding parameters. The CORE method optimises the component weightings for all voxels, ${\left\{z_{n1},z_{n2},\ldots ,z_{nM}\right\}}_{n=1}^{N},$ and global MR properties, $T_{2m}$ and $ADC_{m}$ in this case, using least squares fitting.

### 2.2 InSpect model

Our InSpect algorithm automates spectral mapping, and undertakes data-driven inversion of the Fredholm integral (or Laplace transform). Rather than naively fitting spectra to each voxel independently, the algorithm learns a data-driven low-dimensional representation consistent with the whole image, see bottom panel of Fig. 1 for a visualisation.

The first element of the representation is a pre-specified number, M, of canonical spectral components, $\{F_{1},F_{2},\ldots ,F_{M}\},$ i.e. InSpect infers *spectra* of MR properties, rather than the single values of CORE. These spectral components can consist of a single mode (or *peak*) as in the examples in Fig. 1, or can have multiple modes. Each spectral component has a corresponding voxelwise weight in each N image voxels. Again the weighting of component m in voxel n is denoted $z_{nm},$ so that the full set of voxelwise weights is

(7) $$z_{n}={\left\{z_{n1},z_{n2},\ldots ,z_{nM}\right\}}_{n=1}^{N},\;\text{where}\;0<z_{nm}<1.$$

Note that we relax the condition that these spectral component weights must sum to 1 (as used in (Slator et al., 2020)) to allow for overall changes in scale across voxels.

The signal from each voxel, $S_{n},$ is described by the continuum model of Equation (1), with the effective spectrum in each voxel n given by the weighted sum of the canonical spectral components

(8) $$F\left(z_{n}\right)=\sum_{m=1}^{M}z_{nm}F_{m}$$

where $z_{n}={\left\{z_{nm}\right\}}_{m=1}^{M}$ are the component weights for voxel n. The discrete model for a single voxel is therefore given by rewriting Equation (3)

(9) $$S_{n}=KF\left(z_{n}\right)$$

and we consider Gaussian noise on the observed signal

(10) $$S_{n}∼N\left(KF\left(z_{n}\right),\sigma_{n}^{2}I\right).$$

The log-likelihood across the whole image is therefore

(11) $$\begin{array}{cc} \log\pi ({\left\{S_{n}\right\}}_{n=1}^{N}|F_{1},F_{2},\ldots ,F_{M},z_{n},{\left\{\sigma_{n}^{2}\right\}}_{n=1}^{N}) & =\\ \sum_{n=1}^{N}\log N\left(S_{n};KF\left(z_{n}\right),\sigma_{n}^{2}I\right) \end{array}$$

where N(x;μ,Σ) is the multivariate normal PDF with mean μ and covariance matrix Σ. Note that we assume all observations in a voxel have the same variance, i.e. the covariance matrix is $\sigma_{n}^{2}I$. For simplicity we denote the log-likelihood logπ(D|θ), where $D={\left\{S_{n}\right\}}_{n=1}^{N}$ is the full single- or multi-constrast quantitative MRI dataset (either a single scan or set of scans), and $\theta =\left\{F_{1},F_{2},\ldots ,F_{M},{\left\{z_{n1},z_{n2},\ldots ,z_{nM}\right\}}_{n=1}^{N},{\left\{\sigma_{n}^{2}\right\}}_{n=1}^{N}\right\}$ are the model parameters to be inferred by InSpect.

### 2.3 InSpect inference algorithm

Here we present the InSpect inference algorithm and provide some intuition behind it. The full derivation is given in Supplementary Material. The inference algorithm seeks the parameters θ that maximise logπ(D|θ). In practice, we estimate $\sigma_{n}^{2}$ directly from the data, e.g. for a T2*-diffusion experiment we estimate by calculating the empirical variance of the volumes with b=0 and the lowest TE, and we use the same approach for lower or higher dimensional datasets. The canonical spectral components, $F_{m},$ and voxelwise maps, ${\left\{z_{n1},z_{n2},\ldots ,z_{nM}\right\}}_{n=1}^{N}$ are iteratively optimised by sequentially maximising as follows

1. Initialise the canonical spectral components $\{F_{1},F_{2},\ldots ,F_{M}\},$ by calculating the mean spectrum across the whole image, then assigning the M biggest separate peaks as the initial spectral components. (Naturally, other initialisation strategies are possible.)
2. Initialise the spectral weights for all voxels ${\left\{z_{nm}\right\}}_{n=1}^{N},$ given $\left\{F_{1},F_{2},\ldots ,F_{M}\right\}$
3. Update $F_{m}$ for some m by solving Equation (12) below
4. Update ${\left\{z_{nm}\right\}}_{n=1}^{N},$ subject to $\sum_{m=1}^{M}z_{nm}=1$ by solving Equation (13) for all voxels
5. Repeat steps 3 and 4 until all voxelwise weights have converged according to some tolerance value, or the maximum iteration number is reached.

The update equations are

(12) $$F_{m}=\underset{F_{m}\geq 0}{arg\;min}{∥\sum_{n=1}^{N}\left(Kz_{nm}\right)F_{m}-\sum_{n=1}^{N}\left(S_{n}-K\left(\sum_{m\neq n}z_{nm}F_{m}\right)\right)∥}_{2}^{2}$$

for the spectral components m=1,…,M and

(13) $${\left\{z_{\mathrm{nm}}\right\}}_{m=1}^{M}=\underset{0<z_{\mathrm{nm}}<1}{\arg\;\max}\log N\left(S_{n};KF\left(z_{n}\right),\sigma_{n}^{2}I\right)$$

for the weights in voxels n=1,…,N. Equations (12) and (13) are solved with non-negative least squares and interior-point algorithms respectively.

#### 2.3.1 Model selection to determine the number of canonical spectral components

Finally, we determine the number of canonical spectral components, M. In this paper, we use the Bayesian information criterion (BIC) to guide the choice of M, with a view to automating this process in future iterations of InSpect. For an InSpect run with fixed M, the BIC is

(14) $$\text{BIC}=k\log n-2\log\pi (D|\hat{\theta })$$

where $k=\left(M-1\right)N+M\prod_{m=1}^{M}N_{\omega_{m}}$ is the number of parameters, $n=NN_{s}$ is the number of observations, and $\log\pi (D|\hat{\theta })$ is the maximised value of the log likelihood function, i.e. at the last step of the inference algorithm. The value of M which produces the lowest BIC best explains the data, although we emphasise that other considerations, such as interpretability and prior microstructural knowledge, are also important when choosing the number of canonical spectral components.

## 3 Experiments

### 3.1 Placenta diffusion-relaxometry data

We demonstrate InSpect using previously published placental T2*-diffusion data (Slator et al., 2019b). Whilst this is two-dimensional multi-contrast data, we again emphasise that InSpect is a general method that is also applicable to single- and higher-dimensional datasets. The protocol has 66 diffusion-weightings (ranging from b = 5 to 1600 s mm${}^{-2},$ including six b = 0 volumes) and 5 TEs (78, 114, 150, 186, 222 ms) for a total of 330 contrast-encodings. Other acquisition parameters were FOV = 300 × 320×84 mm, TR = 7 s, SENSE = 2.5, halfscan = 0.6, resolution = 3mm${}^{3}$. We considered 13 scans from 12 women, of whom 9 were categorised as healthy controls, two had chronic hypertension in pregnancy, and one had pre-eclampsia (PE) with additional fetal growth restriction (FGR). One participant with chronic hypertension was scanned two times, four weeks apart, and developed superimposed pre-eclampsia by the second scan (see Table 1 for full participant details). An ROI comprising the whole placenta and the adjacent section of uterine wall was manually segmented on all images.

**Table 1:** Participant details.

Participant ID	GA at scan (weeks)	Cohort
1	23.72	Control
2	23.86	Control
3	25.43	Control
4	25.72	Control
5	26.14	Control
6	26.72	Control
7	27.14	Control
8	28.29	Control
9	28.86	Control
10	34.43	CH
11	27.7	PE+FGR
12 (scan 1)	30.71	CH
12 (scan 2)	34.14	CH+PE

GA - gestational age, PE - pre-eclampsia, CH - chronic hypertensive, FGR - fetal growth restriction

There are two main approaches possible when applying InSpect to a dataset with multiple scans: run on each MRI scan independently and find separate canonical spectra and mappings for each; or run on all scans simultaneously and estimate a common set of spectra. The best approach will depend on the specific application. One important consideration is the extent to which one wants to probe within-image heterogeneity, as opposed to across-image differences. In this paper our aim is to gain an initial idea of typical placental T2*-ADC spectra, and their spatial distributions, across healthy and unhealthy tissue. We therefore run InSpect on the data for all patients simultaneously - i.e. fitting to all images jointly, estimating a common set of spectral components across all participants. We ran InSpect, including calculating the BIC, for all choices of the number of components from M=2 to M=10. We terminated the algorithm when voxelwise spectral weight maps had converged to within a tolerance of $10^{-3},$ or after five iterations were completed.

### 3.2 Simulations

**Fig. 2:**
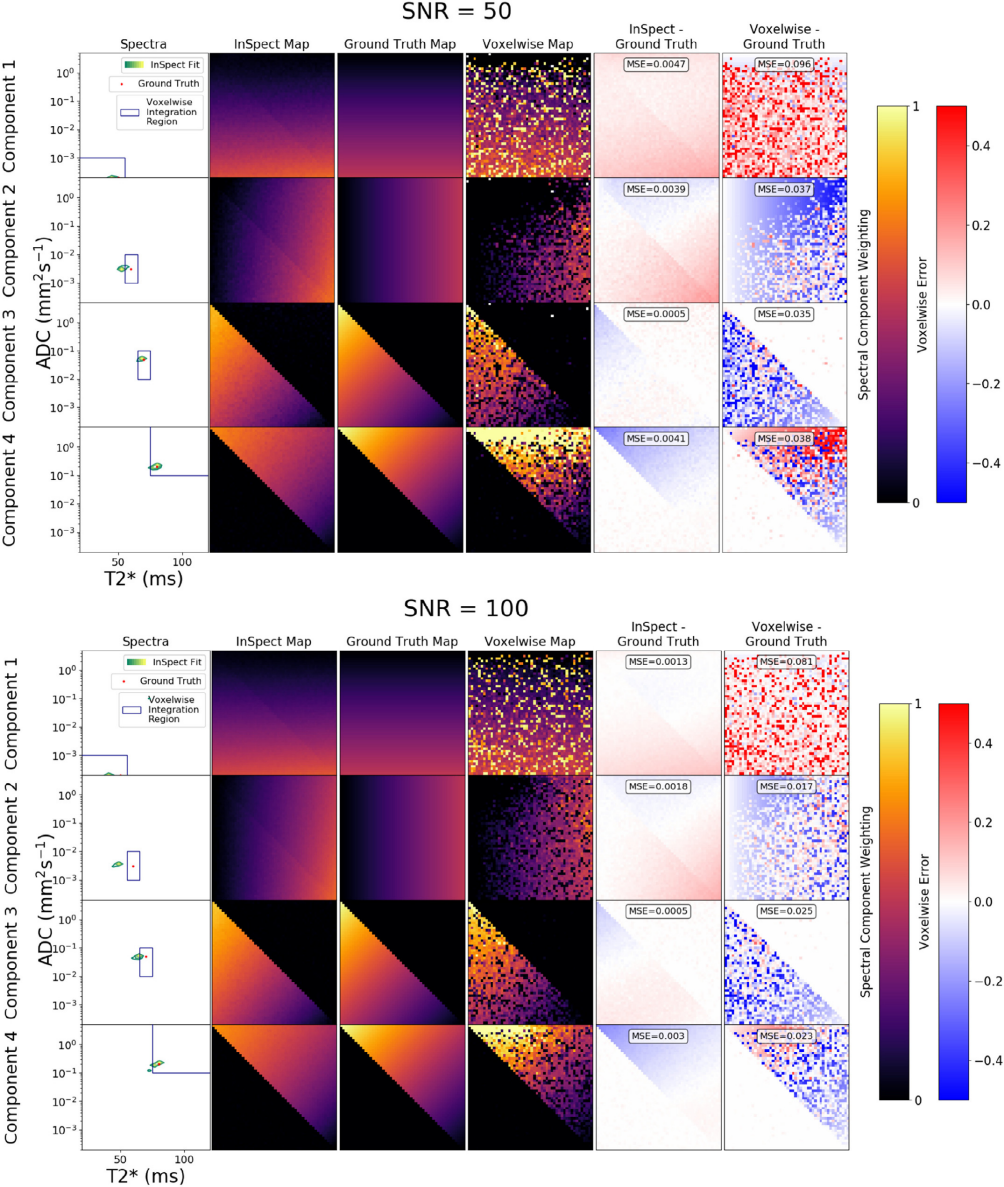
InSpect applied to simulated images for two noise levels. For each SNR, the InSpect output is presented in columns 1–2: inferred spectral components (green-yellow peaks, column 1), and corresponding voxelwise weights (column 2). Column 3: ground truth voxelwise weights; ground truth spectral components (red dots in column 1) have fixed T2*-ADC values of (0.05, 0.0002), (0.06, 0.003), (0.07, 0.05) and (0.08 ms, 0.2 mm2/s). Column 4: maps obtained by numerical integration (within blue regions of column 1) of voxelwise spectral fits (we set α=0.01 for these fits, following (Hansen, 1992)). Columns 5 and 6: difference and mean square errors between ground truth and inferred maps. See Fig. S1 for corresponding maps for simulations with higher SNR. (For interpretation of the references to colour in this figure legend, the reader is referred to the web version of this article.)

We also tested InSpect on simulated diffusion-relaxometry data. Four synthetic canonical spectral components - informed by observed placental spectra (Slator et al., 2019b) - were first defined (first columns, Fig. 2). We next defined ground truth voxelwise weights on a 50-by-50 image (i.e. 2500 voxels total, see third columns in Fig. 2). Given these we simulated diffusion-relaxometry scans as

(15) $$S_{n}\left(b,T_{E}\right)=KF\left(z_{n}\right)=K\left(\sum_{m=1}^{M}z_{nm}F_{m}\right)$$

using the same b-values and TEs as the placental data. We normalised each voxel’s data so that the mean of the volumes with b=0 and the lowest TE equalled 1. We added Rician noise with SNR from 50 to 400 - this is comparable to the placenta data where we calculated SNRs ranging from 50 to 200. We ran InSpect for all choices of the number of components from M=2 to M=8 on these scans. Equivalently to the placenta case, we terminated the algorithm when the weights had converged to within a tolerance of $10^{-3},$ which typically took around three iterations for the simulated data. We also fit voxelwise T2*-ADC spectra to all scans by solving Equation (4), with α set to 0.01 using the L-curve method (Hansen, 1992). We hence derived spectral volume fraction maps by integrating voxelwise spectra in four regions of T2*-ADC space (i.e. sROIs) as in (Mackay, Whittall, Adler, Li, Paty, Graeb, 1994, Kim, Doyle, Wisnowski, Kim, Haldar, 2017, Benjamini, Basser, 2017). We chose these sROIs based on the ground truth components, which we note would not be possible on real data.

## 4 Results

**Fig. 3:**
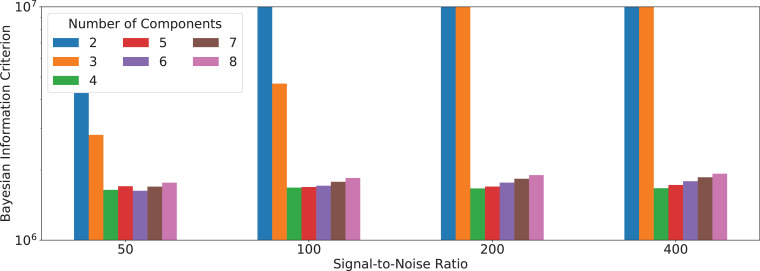
Bayesian information criterion for InSpect run on simulations for various choices of M, the number of canonical spectral components. For clarity the y-axis is limited to between $10^{6}$ and $10^{7}$. Simulation parameters as Fig. 2.

**Fig. 4:**
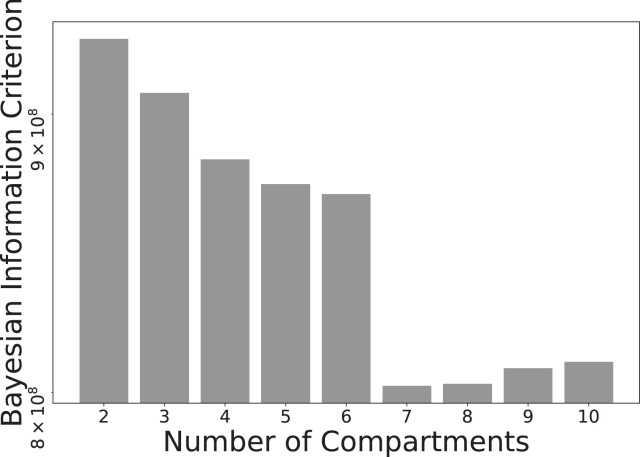
Bayesian Information Criterion for InSpect run on all placenta scans for various choices of M, the number of canonical spectral components. Fits with lower BIC values explain the data better.

**Fig. 5:**
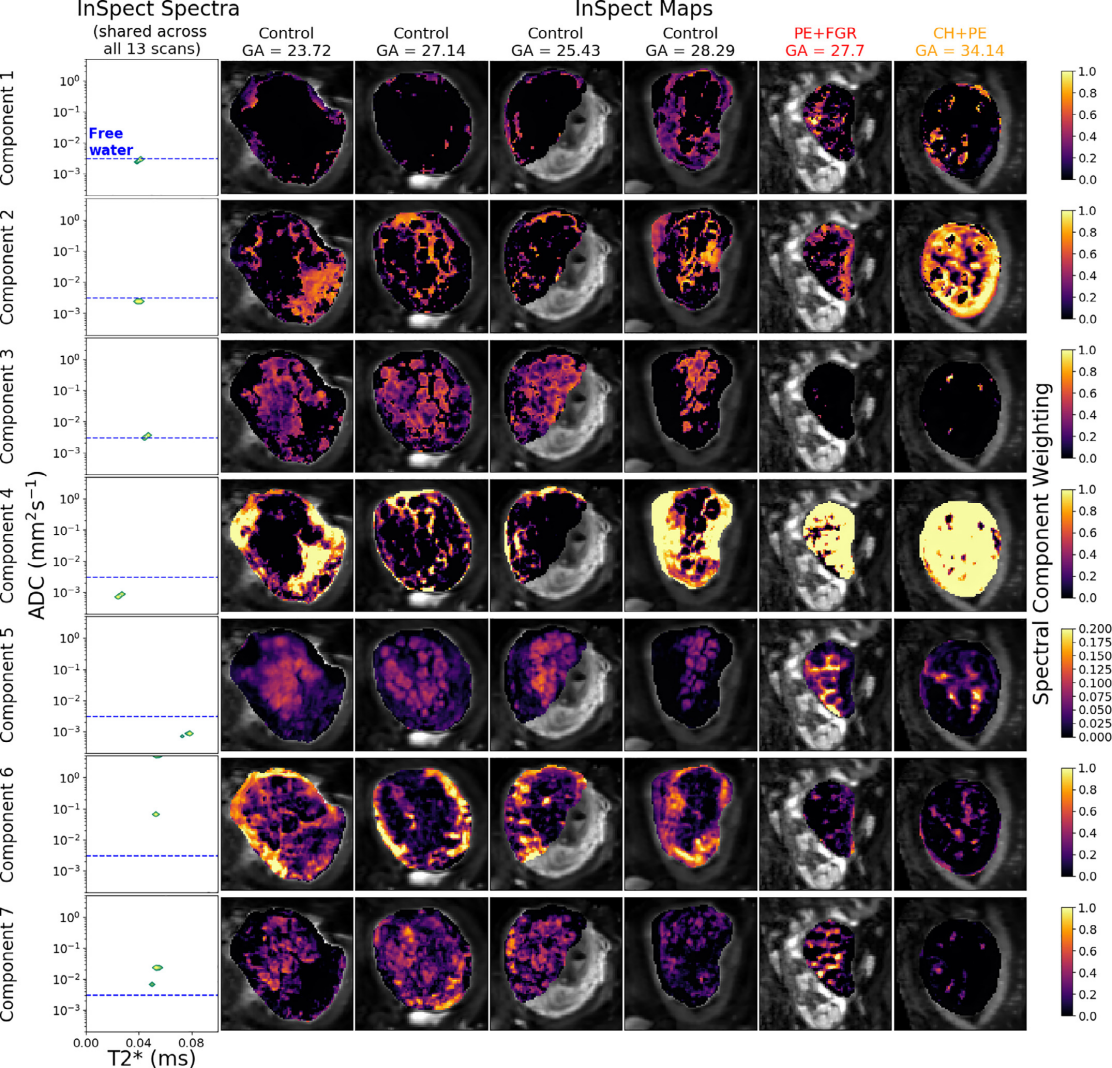
Seven-component InSpect run on 13 placenta diffusion-relaxometry scans. The leftmost boxes display the canonical spectral components, shared across all 13 participants. The remaining boxes show the corresponding component weighting maps for 6 of the 13 scans, with each column displaying a single participant’s maps (see Fig. 6 for the remaining 7 maps). Two participants had been diagnosed with a pregnancy complication at scan time (red outline). Note that the final columns of this Figure and Fig. 6 display maps for the same participant, scanned twice, four weeks apart. See (Slator et al., 2019b) for corresponding maps calculated with a voxelwise spectral approach. (For interpretation of the references to colour in this figure legend, the reader is referred to the web version of this article.)

**Fig. 6:**
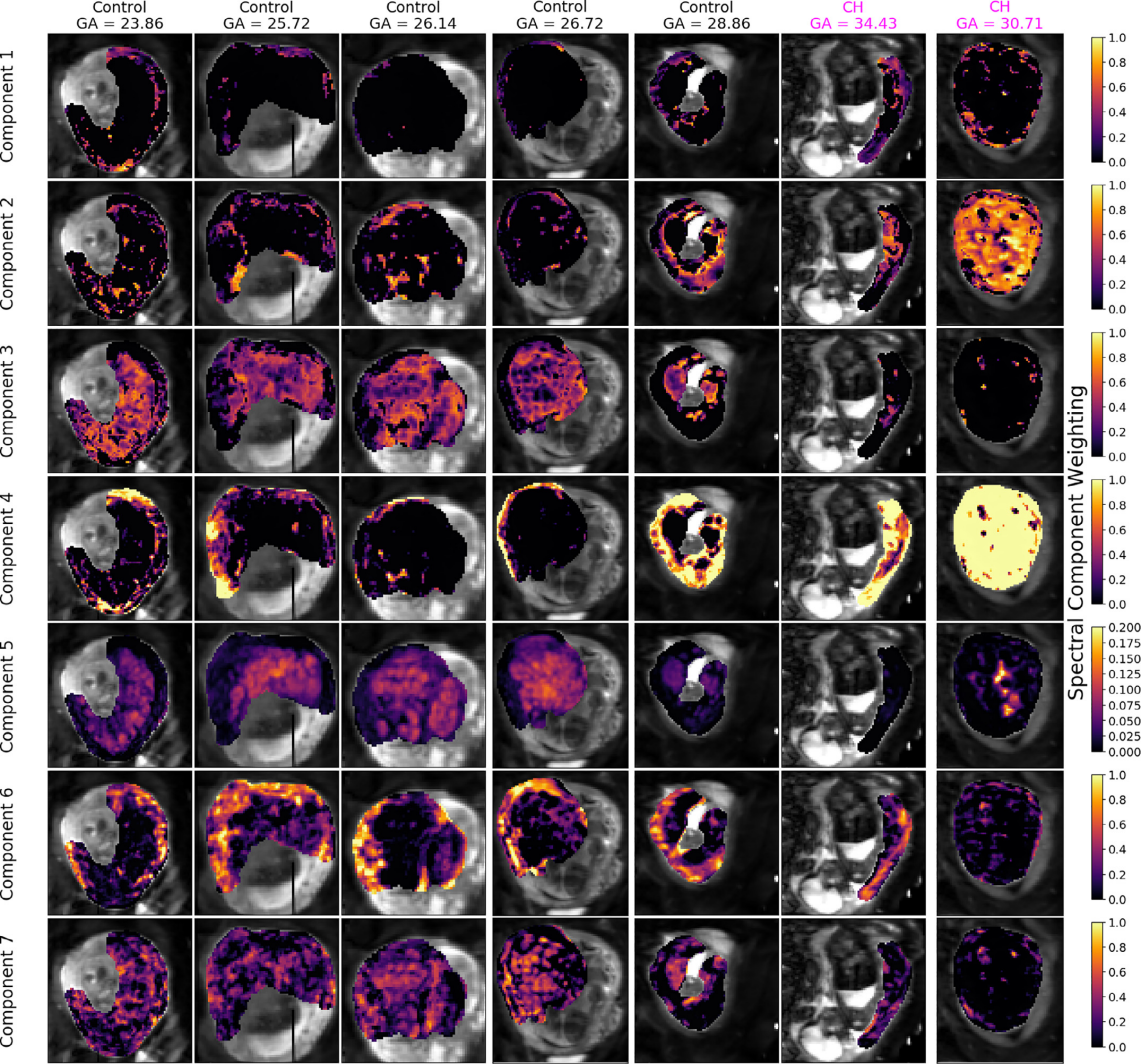
InSpect maps for 7 participants from the seven-component InSpect run on 13 placenta diffusion-relaxometry scans. Each row displays maps for a single canonical spectral component - see first column of Fig. 5 for the corresponding spectra. Columns display the maps for a single scan. Note that the final column in Figs. 5 and 6 display maps for the same participant, scanned twice.

**Fig. 7:**
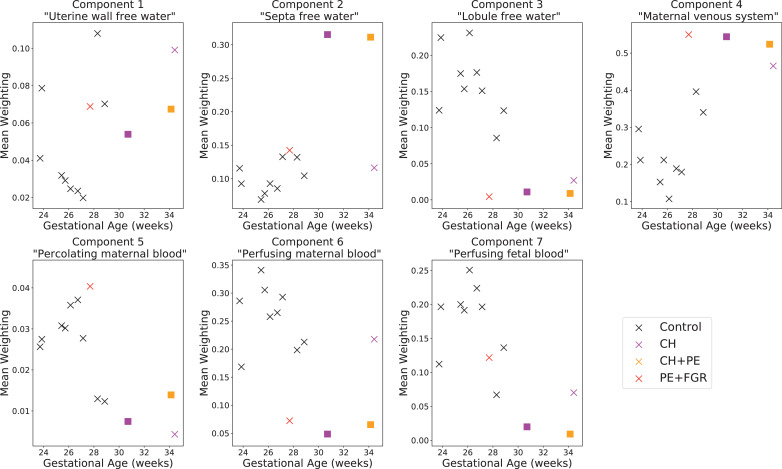
Scatterplot of mean values of spectral component weightings in placenta and uterine wall ROI against gestational age. Square markers indicate the single participant who was scanned twice.

Figs. 2 and 3 demonstrate InSpect applied to simulated diffusion-relaxometry images, and Figs. 4, 5 and 6 present the simultaneous InSpect run on all participants’ placental MR images. Fig. 7 displays how InSpect parameters in the placenta change over gestation.

Figs. 2 and S1 demonstrate that InSpect dramatically outperforms the voxelwise approach on simulated data. At all noise levels InSpect captures the core features of the maps, and more accurately recovers the ground truth than voxelwise maps. There is a modest diagonal artifact in component 1 and 2 InSpect maps, most noticeable at lower SNRs, which arises because of the large difference in component 3 and 4 weightings on either side of this line slightly biasing component 1 and 2 weight estimation. At SNR=400 (Fig. S1) InSpect maps are almost identical to the ground truth, whereas the voxelwise spectral integration maps remain somewhat noisy. InSpect also accurately recovers ground truth spectral components (first columns, Fig. 2), we emphasise that it is not possible to automatically recover these components using the standard voxelwise approach - rather corresponding regions of the spectral domain are manually defined.

Fig. 3 shows that the BIC correctly suggests that choosing M=4 components best explains the simulated data at all noise levels, except for one anomaly at SNR=50 where M=6 has a very slightly lower BIC value.

Fig. 4 shows that InSpect with M=7 components had the lowest BIC value for the placenta scans, demonstrating that this choice is optimal in an information theoretic sense. We emphasise that this is not necessarily the “best” choice - a smaller choice of M may make the InSpect algorithm output more readily interpretable in practice.

Figs. 5 and 6 display the seven-component InSpect run on all participants’ placental MR images simultaneously. The first column in Fig. 5 shows the canonical spectral components (which are shared across all participants), and the remaining columns show the corresponding weighting maps. The seven canonical spectral components have distinct characteristics and - even though the algorithm imposes no direct anatomical analogue for any of the components - the corresponding maps identify clear anatomical structures which are consistent across control placentas, and show clear differences in dysfunctional placentas. This suggests that the canonical spectral components are potentially identifying distinct tissue environments, and that those tissue environments could be salient to placental dysfunction.

Given the observed spatial patterns and corresponding canonical spectral component characteristics in placental data, we can make initial speculations about the microstructural and microcirculatory environments associated with each component, although we emphasise that whilst some signal components appear to latch on to specific tissue structures, they cannot be fully interpreted in this way as nothing in the InSpect algorithm guarantees this specific mapping:

• Components one, two and three all consist of a single spectral peak with ADC close to free water. In weighting maps, component one appears more prominent in the uterine wall, component two appears in the areas surrounding placental lobules, and component three appears in the centre of placental lobules. These observations are consistent with these components representing freely diffusing water in the uterine wall, septa, and lobules respectively.
• Component four has a single peak with low ADC and low T2*, and its maps show high weighting in areas of maternal tissue. This is consistent with this component representing water with lower oxygen saturation and diffusion hindered by tissue structures, potentially the maternal venous system.
• Component five has a single peak with low ADC and high T2*, and is spatially prominent in lobular patterns within the placenta. These observations are consistent with this component representing water with high oxygen saturation whose diffusion is hindered, such as oxygenated maternal blood percolating through convoluted intervillous space (e.g. (Dellschaft et al., 2020)).
• Component six has two peaks with ADC much higher than free water, and reasonably high T2*. It is spatially prominent in the uterine wall. This suggests that this component reflects maternal blood perfusing in maternal vasculature.
• Component seven has two peaks with ADC higher than free water, and is spatially prominent in lobular patterns in the placenta. This suggests that this component reflects fetal blood perfusing in fetal vasculature.

To make an initial assessment of InSpect’s ability to quantify and predict changes during development and disease, we plotted the mean canonical spectral component weightings in the placenta and uterine wall ROI for all participants across gestation (Fig. 7).

Given our speculative assignments of components to tissue environments, the trends we observe are consistent with known placental structure changes across gestation and in disease:

• Component three (“free water in placental lobules”) and component five (“maternal blood in intervillous space”) decrease over gestational age; this is consistent with terminal villi growth shrinking the size of maternal blood pools (Jackson et al., 1992).
• The perfusion-associated components six and seven both decrease over gestational age. This is consistent with the observed decrease in diffusion MRI-derived perfusion fraction over gestational age (Slator, Hutter, Panagiotaki, Rutherford, Hajnal, Alexander, Hutter, Panagiotaki, Rutherford, Hajnal, Alexander, 2018, Anderson, Hansen, Haals, Sinding, Petersen, Frøkjær, Peters, Sørensen, 2020).
• Component four (“maternal venous system”) is significantly higher in dysfunctional placentas compared to controls, potentially reflecting lower blood oxygenation (Derwig, Lythgoe, Barker, Poon, Gowland, Yeung, Zelaya, Nicolaides, 2013, Sinding, Peters, Frøkjaer, Christiansen, Petersen, Uldbjerg, Sørensen, 2016, Sinding, Peters, Frøkjær, Christiansen, Petersen, Uldbjerg, Sørensen, 2017, Sinding, Peters, Poulsen, Frøkjær, Christiansen, Petersen, Uldbjerg, Sørensen, 2018, Ingram, Morris, Naish, Myers, Johnstone, 2017, Hutter, Slator, Jackson, Gomes, Ho, Story, O’Muircheartaigh, Teixeira, Chappell, Alexander, Rutherford, Hajnal, 2019, Ho, Hutter, Jackson, Seed, Mccabe, Al-Adnani, Marnerides, George, Story, Hajnal, Rutherford, Chappell, 2020).
• Component six (“perfusing maternal blood”) is significantly lower in dysfunctional placentas compared to controls, potentially reflecting reduced perfusion (Derwig, Lythgoe, Barker, Poon, Gowland, Yeung, Zelaya, Nicolaides, 2013, Sohlberg, Mulic-Lutvica, Lindgren, Ortiz-Nieto, Wikström, Wikström, 2014, Sohlberg, Mulic-Lutvica, Olovsson, Weis, Axelsson, Wikström, Wikström, 2015, Anderson, Hansen, Haals, Sinding, Petersen, Frøkjær, Peters, Sørensen, 2020).

## 5 Discussion

We introduce and demonstrate InSpect, a data-driven algorithm for continuous mapping of spectral components in single- or multi-contrast quantitative MRI experiments. The approach addresses key limitations of traditional approaches, which are unstable with standard MRI noise levels and require manual spectral labelling to obtain parametric maps. Specifically, InSpect exploits the redundancy in spectral variation across individual (or groups of) images, and automates mapping of spectral components.

On simulated data we show that InSpect dramatically outperforms the standard voxelwise approach, even when the total number of voxels is relatively small compared to a typical clinical scan (Fig. 2). On placental diffusion-relaxometry MRI data InSpect maps clearly show anatomical structures (Figs. 5 and 6). These maps and the properties of their corresponding spectral components reveal some association of InSpect components with known tissue structures. These associations are consistent with the observed patterns across gestation (Fig. 7), and reveal insights that are consistent with known placenta microstructure and microcirculation properties, in healthy controls and participants with pregnancy complications.

Components one, two, and three have very similar spectral properties - a single peak with ADC close to free water - but maps that are prominent in the uterine wall, septa, and lobules respectively. Compared to component one, component two has a slightly lower ADC, this is consistent with the higher ADC values typically seen in the uterine wall (e.g. (Slator et al., 2019b)). Component three has a slightly higher T2* than component one, this is consistent with the higher T2* values seen in the centre of the lobule-like structures (e.g. (Hutter et al., 2019)). These observations suggest that although their spectra appear very similar, these three components could encode subtle microstructural changes affecting the diffusion of free water across the uterine wall, septa, and lobules.

Components six (“perfusing maternal blood”) and seven (“perfusing fetal blood”) both have two peaks with ADC above free water, although component six peaks have markedly higher ADCs than seven. Component six is prominent in the uterine wall, whereas component seven is prominent in the placenta. These observations are consistent with the higher values seen in the uterine wall in ADC maps (Slator, Hutter, McCabe, Gomes, Price, Panagiotaki, Rutherford, Hajnal, Alexander, 2018, Slator, Hutter, Palombo, Jackson, Ho, Panagiotaki, Chappell, Rutherford, Hajnal, Alexander, Hutter, Slator, Jackson, Gomes, Ho, Story, O’Muircheartaigh, Teixeira, Chappell, Alexander, Rutherford, Hajnal, 2019). The possibility that these maps could separately quantify maternal and fetal circulations, similarly to (Melbourne, Aughwane, Sokolska, Owen, Kendall, Flouri, Bainbridge, Atkinson, Deprest, Vercauteren, David, Ourselin, Aughwane, Mufti, Flouri, Maksym, Spencer, Sokolska, Kendall, Atkinson, Bainbridge, Deprest, Vercauteren, Ourselin, David, Melbourne, 2020), has potential applications in identifying placental insufficiency.

Components three (“lobule free water”), four (“maternal venous system”), and six (“perfusing maternal blood”) show the clearest differences in dysfunctional placentas, likely indicative of pathology, and are hence promising for predicting and diagnosing pregnancy complications. However, we need to increase participant numbers, particularly by scanning healthy controls across a wider distribution of gestational ages, in order to fully evaluation the predictive and diagnostic power of these metrics.

Whilst it is tempting, as we have speculated above for the placenta, to think of InSpect components as representing distinct “tissue types”, there are many potential inaccuracies and uncertainties. The relationship between the two will likely be disrupted by various effects, and so we emphasise again the need for caution in lieu of any direct validation of these links. We also emphasise that we cannot definitively know the spatial scales of these structures. They could be bulk “tissue types” that contain multiple microstructural compartments, e.g. maternal placenta, fetal placenta; or more specific “tissue compartments”, e.g. intracellular, extracellular, fetal blood, maternal blood. The scale of the mapped structures will depend on the number of InSpect components - less components will tend to output maps of tissue types consisting of multiple tissue compartments, whereas more components will disentangle these tissue types into single tissue compartments. The long-term hope is that InSpect components are close enough to specific tissue structures that they can reveal pathology with greater specificity than indices that do not attempt to disentangle tissue types.

### 5.1 Relationship to other methods

InSpect offers a new way of mapping single- or multi-dimensional quantitative MRI data that is complementary to existing methods based on spatial regularisation (Kim et al., 2017a) or marginal distributions (Benjamini and Basser, 2016). Unlike these methods, InSpect does not require manual identification of sROIs to obtain parametric maps, avoiding this problem through data-driven regularisation. CORE NMR (Stilbs, Paulsen, Griffiths, 1996, Stilbs) was a successful first attempt at regularisation of similar inverse problems in a data-driven way. Whilst InSpect and CORE share conceptual similarities, we note the following advances and novelties:

• CORE infers single fixed values for the underlying MR properties of each component, InSpect infers spectra across these properties. This allows InSpect to capture components with more complex diffusion, relaxation, or diffusion-relaxation profiles than a single fixed value, which can reflect more intricate tissue environments.
• InSpect can automatically select the number of spectral components, whereas CORE uses a fixed number of components.
• As opposed to CORE, InSpect uses a general log-likelihood formulation. This framework accommodates more accurate MRI noise models, e.g. Rician, in future.
• The differences in model formulation lead to different optimisation procedures. InSpect uses a modified EM algorithm to infer component spectra (M-step) and their associated weightings (E-step). On the other hand, CORE uses a multi-level least squares optimisation to infer the global MR properties (top level) and component weightings (lower level).

Our method is also comparable to blind source separation (BSS) techniques (e.g. Kim et al. (2015) and Molina-Romero et al. (2018)). The main advantage of InSpect over BSS is that we incorporate a well-defined basic MRI model, allowing us to explicitly reconstruct signal components that we can associate with distinct tissue compartments. However, BSS would be more appropriate when explicit signal models are unknown or inaccurate.

Moreover, InSpect is complementary to image denoising techniques, such as Marchenko-Pastur distribution-based principal component analysis (MP-PCA) (Veraart et al., 2016), which has been shown to improve voxelwise mapping of multi-exponential relaxometry data (Does et al., 2019). This PCA-based denoising technique could be adapted in future to spectral mapping of quantitative MRI data by using an InSpect-like kernel instead of MP.

In future, we will undertake an extensive comparison of all these techniques, whilst carefully considering how to compare methods fairly as this is not a clearly defined standalone task - so the optimal technique will depend on the specific application.

### 5.2 Limitations and future work

There are limitations to our algorithm in its current form that motivate potential improvements. Firstly, although we demonstrated choosing the number of canonical spectral components by running InSpect with several values of M, then determining the final M with the BIC, this approach may not be optimal in all cases. Running InSpect with fewer components is less computationally expensive and post-processing will be simpler, although it is not immediately clear which approach is better for interpretation. To explore this, we plotted output from all InSpect runs on the placental data with M=2 to M=6 components (Figs. S2 to S6 in Supplementary Material). We note that for InSpect runs with less than six components (i.e. Figs. S2 to S5, and also (Slator et al., 2020) where we used four components) there are not two clearly separated perfusion components. Therefore, for these fits, it is not possible to putatively assign two components to maternal and fetal perfusion, as for components six and seven in the seven-component fit. Moreover, in runs with lower M we typically recover spectral components which have multiple peaks, so likely consist of multiple tissue microenvironments, whereas for M=7 most components have a single peak, so are more likely to represent a single microstructural environment. In this sense the seven-component run is easier to interpret. However, it may be the case that less than seven components are sufficient to make the necessary diagnostic or prognostic observations from the data - we will test this hypothesis extensively in future. We will also explore other ways of selecting the number of components, such as quantifying the distance between estimated maps (or spectra) with an appropriate image (or distribution) similarity measure then thresholding in order to set an upper limit on M, and model selection via cross validation. Users may also have a fixed number of target microstructural compartments in mind, so it may be beneficial to leave the possibility of manually defining M.

In this study, we show that InSpect spectral components appear to be associated with distinct anatomical features in the placenta. In future, depending on the specific application, we could constrain the spectra to ensure sensitivity and specificity to anatomical features of interest. Another option is to incorporate a Markov random field (similar to (Marinescu et al., 2019)) into the weighting maps to make it more likely that anatomical features are picked up.

Here we ran InSpect on groups of placental MRI scans simultaneously. This is the obvious approach for group studies since the components need to be consistent across images/individuals, but may average over some important within-individual features. In future we will compare to individual scan fits, with focus on which approach best differentiates controls from disease. This choice also impacts on downstream analysis, for example if a dataset is extended, it may not be straightforward to compare InSpect results for the extended dataset with the original dataset (in the group fitting case). There is hence scope for substantial further work to assess the consistency of components and model selections across different instances of datasets - a bootstrap or cross-validation analysis would likely be very informative.

Since it assumes a high level of redundancy across images, Inspect will likely latch onto the strongest spectral components and may miss important components that appear in only a small number of voxels. This could potentially be addressed by running InSpect with a varying number of spectral components - inferring aggregate information from the fits with few components and finer details from the many component fits, or alternatively by optimising a different objective function that strongly emphasises regions of the image that are poorly reconstructed.

Our algorithm assumes that underlying spectral components are fixed. We tested the robustness of InSpect to voxelwise variations in ground truth spectra by running simulations with additional noise. Specifically, we introduced voxelwise normally-distributed perturbations to the ADC and T2* values of the four synthetic canonical spectral components seen in Figs. 2 and S1. Figs. S7 and S8 display the resulting maps, which are noisier but still recover the salient structures, and show that InSpect still dramatically outperforms the voxelwise approach. In future we will explore more general approaches for increasing InSpect’s robustness to voxel-by-voxel noise, such as hierarchical modelling that explicitly accounts for variation in the MR properties of the underlying spectra.

Although we applied to two-dimensional T2*-diffusion data in this paper, our InSpect formulation is agnostic to the dimensionality. Applying the framework to single-contrast quantitative MRI is particularly attractive, since there are applications in many tissue types and imaging modalities. Our approach could enable significantly improved mappings compared to voxelwise model fitting methods. For example, the framework is immediately applicable to spectral analysis of multi-echo T2 relaxometry for myelin water imaging in the brain (Alonso-Ortiz, Levesque, Pike, 2015, Nagtegaal, Koken, Amthor, Bresser, Vos, Doneva, 2020), and luminal water imaging in the prostate (Sabouri, Chang, Savdie, Zhang, Jones, Goldenberg, Black, Kozlowski, 2017, Devine, Giganti, Johnston, Sidhu, Panagiotaki, Punwani, Alexander, Atkinson, 2019). Whilst we focused on diffusion-relaxometry experiments, InSpect also generalises to other multidimensional spectral techniques, such as the range of “diffusion-X” spectral techniques (Callaghan, 1991), relaxometry-relaxometry (English et al., 1991), and diffusion exchange spectroscopy (Benjamini, Komlosh, Basser, 2017, Breen-Norris, Siow, Walsh, Hipwell, Hill, Roberts, Hall, Lythgoe, Ianus, Alexander, Walker-Samuel, 2020).

InSpect reduces the SNR necessary to estimate and map spectral features using continuum models and quantitative MRI data. The minimum SNR limit for data reconstruction could be further lowered by accounting for Rician noise. The method can hence underpin novel scanning methods for obtaining new data, by leveraging the SNR gain to reduce scanning times and/or improve spatial resolution, ultimately increasing the likelihood of translating quantitative MRI spectroscopy to clinical applications.

## 6 Conclusion

We present a data-driven approach for quantitative MRI data analysis, and demonstrate its ability to quantify placental dysfunction. InSpect exploits within-image redundancies to simultaneously estimate a set of canonical spectral components and their mapping across images, offering significant advantages over typical single- and multidimensional spectrum estimation methods. The method makes quantitative MRI spectroscopy possible in a wide range of new application areas across tissue types and imaging modalities.

## Funding

This work was supported by the NIH Human Placenta Project grant 1U01HD087202-01 (Placenta Imaging Project [PiP]); Wellcome Trust (201374/Z/16/Z); EPSRC (N018702, M020533, EP/N018702/1); NIHR (RP-2014-05-019); the National Institute for Health Research (NIHR) Biomedical Research Centre at University College London Hospitals NHS Foundation Trust and University College London; the Wellcome EPSRC Centre for Medical Engineering at Kings College London (WT 203148/Z/16/Z) and by the NIHR Biomedical Research Centre based at Guys and St Thomas NHS Foundation Trust and Kings College London. PJS and DCA were funded by the 10.13039/501100000780European Unions Horizon 2020 research and innovation programme under grant agreement No. 666992. The views expressed are those of the authors and not necessarily those of the NHS, the NIHR or the Department of Health.

## CRediT authorship contribution statement

**Paddy J. Slator:** Writing - original draft, Writing - review & editing, Conceptualization, Methodology, Software, Formal analysis. **Jana Hutter:** Methodology, Conceptualization, Data curation, Writing - review & editing. **Razvan V. Marinescu:** Conceptualization, Writing - review & editing. **Marco Palombo:** Writing - review & editing, Conceptualization. **Laurence H. Jackson:** Methodology, Data curation. **Alison Ho:** Data curation, Resources. **Lucy C. Chappell:** Data curation, Resources, Funding acquisition. **Mary Rutherford:** Conceptualization, Funding acquisition. **Joseph V. Hajnal:** Conceptualization, Funding acquisition. **Daniel C. Alexander:** Conceptualization, Writing - review & editing, Funding acquisition.

## Declaration of Competing Interest

The authors declare that they have no known competing financial interests or personal relationships that could have appeared to influence the work reported in this paper.

## Appendix A Supplementary materials

Supplementary Data S1 Supplementary Raw Research Data. This is open data under the CC BY license http://creativecommons.org/licenses/by/4.0/ Supplementary Data S1
