# Supplementary material for: Data-Driven multi-Contrast spectral microstructure imaging with InSpect: INtegrated SPECTral component estimation and mapping
Source: Med Image Anal. 2021 Jul;71:102045. doi: 10.1016/j.media.2021.102045 (PMC8543043; doi:10.1016/j.media.2021.102045)
Supplement: Supplementary Data S1 — Supplementary Raw Research Data. This is open data under the CC BY license http://creativecommons.org/licenses/by/4.0/ [file mmc1.pdf]

Supplementary Material

6.1. InSpect Inference Algorithm Derivation

To derive maximisation steps for the canonical spectral components $\{F_m\}_{m=1}^{M}$, and voxelwise weights, $\{z_{nm}\}_{n=1}^{N}$ we rearrange the log-likelihood (Equation (11)) by first noting that

$$\mathcal{N}\left(\mathbf{S}_n; \mathbf{K}F(\mathbf{z}_n), \sigma_n^2 I\right) = \prod_{k=1}^{N_s} \frac{1}{\sqrt{2\pi\sigma_n^2}} \exp\left(-\frac{\left(S_n^{(k)} - KF(\mathbf{z}_n)^{(k)}\right)^2}{2\sigma_n^2}\right) \tag{16}$$

$$= \frac{1}{\sqrt{2\pi\sigma_n^2}} \exp\left(-\frac{1}{2\sigma_n^2}\sum_{k=1}^{N_s}\left(S_n^{(k)} - KF(\mathbf{z}_n)^{(k)}\right)^2\right) \tag{17}$$

$$= \frac{1}{\sqrt{2\pi\sigma_n^2}} \exp\left(-\frac{1}{2\sigma_n^2}\left\|\mathbf{S}_n - \mathbf{K}F(\mathbf{z}_n)\right\|_2^2\right) \tag{18}$$

and hence

$$\log \pi(\mathbf{D}|\theta) = \sum_{n=1}^{N} \log \mathcal{N}\left(\mathbf{S}_n; \mathbf{K}F(\mathbf{z}_n), \sigma_n^2 I\right) \tag{19}$$

$$= \sum_{n=1}^{N} -\frac{1}{2}\log 2\pi\sigma_n^2 - \frac{1}{2\sigma_n^2}\left\|\mathbf{S}_n - \mathbf{K}F(\mathbf{z}_n)\right\|_2^2 \tag{20}$$

6.1.1. Maximisation step for canonical spectral components

We first derive a maximisation step for a canonical spectrum, F_j conditioned on the voxelwise weights $\{\mathbf{z}_n\}_{n=1}^{N}$, and the other spectra $\{F_m\}_{m\neq j}$. For the canonical spectral components, $\mathbf{F}_1, ..., \mathbf{F}_M$, we need to solve

$$F_k = \underset{F_k\geq 0}{\arg\max} \log \pi(\mathbf{D}|\theta) \tag{21}$$

$$= \underset{F_k\geq 0}{\arg\max} \sum_{n=1}^{N} -\frac{1}{2}\log 2\pi\sigma_n^2 - \frac{1}{2\sigma_n^2}\left\|\mathbf{S}_n - \mathbf{K}F(\mathbf{z}_n)\right\|_2^2 \tag{22}$$

$$= \underset{F_k\geq 0}{\arg\min} \sum_{n=1}^{N} \frac{1}{2\sigma_n^2}\left\|\mathbf{S}_n - \mathbf{K}F(\mathbf{z}_n)\right\|_2^2 \tag{23}$$

$$= \underset{F_k\geq 0}{\arg\min} \sum_{n=1}^{N} \frac{1}{2\sigma_n^2}\left\|\mathbf{K}z_{nk}F_k - \left(\mathbf{S}_n - \mathbf{K}\sum_{m\neq k} z_{nm}F_m\right)\right\|_2^2 \tag{24}$$

Taking the derivation of the function with respect to F_k (in numerator layout), and setting equal to zero gives

$$\mathbf{0}_{1,N_{\omega_1}N_{\omega_2}} = \sum_{n=1}^{N} \frac{1}{\sigma_n^2}\left(\mathbf{K}z_{nk}F_k - \left(\mathbf{S}_n - \mathbf{K}\sum_{m\neq k} z_{nm}F_m\right)\right)^T \mathbf{K}z_{nk} \tag{25}$$

where $\mathbf{0}_{1,N_{\omega_1}N_{\omega_2}}$ is the zero (row) vector of length $N_{\omega_1}N_{\omega_2}$. Here we have used the facts that, in numerator layout, if x is a column vector then $\frac{d}{dx}\frac{1}{2}\|x\|_2^2 = x^T$, and if $y = KF$ (K is n by m, F is m by 1), then $dy/dF = K$. Equation (25) is satisfied if

$$\mathbf{0}_{1,N_s} = \mathbf{K}z_{nk}F_k - \left(S_n - \mathbf{K}\sum_{m\neq k} z_{nm}F_m\right) \tag{26}$$

for all n voxels, or equivalently

$$\mathbf{0}_{1,N_s} = \sum_{n=1}^{N} \mathbf{K}z_{nk}F_k - \left(S_n - \mathbf{K}\sum_{m\neq k} z_{nm}F_m\right) \tag{27}$$

Rearranging to get the F_k terms on the LHS gives

$$\left(\sum_{n=1}^{N} \mathbf{K}z_{nk}\right) F_k = \sum_{n=1}^{N}\left(\mathbf{S}_n - \mathbf{K}\sum_{m\neq k} z_{nm}F_m\right). \tag{28}$$

So we can calculate the canonical spectrum components by modifying Equation (4) as follows

$$\mathbf{F}_k = \underset{\mathbf{F}_k\geq 0}{\arg\min} \left\|\left(\sum_{n=1}^{N} \mathbf{K}z_{nk}\right)\mathbf{F}_k - \sum_{n=1}^{N}\left(\mathbf{S}_n - \mathbf{K}\left(\sum_{m\neq k} z_{nm}\mathbf{F_m}\right)\right)\right\|_2^2 \tag{29}$$

In other words, the dictionary, K, in Equation (4) is replaced with a voxelwise weighted version $\sum_{n=1}^{N}\mathbf{K}z_{nk}$. And the signal $\mathbf{S}$ is replaced by the weighted sum across all voxels of the signal minus the contribution from the other spectrum components, $\{\mathbf{F}_m\}_{m\neq k}$. We can solve Equation (29) with non-negative least squares regression as described earlier.

6.1.2. Estimation of voxelwise canonical spectral component weights

To find the maximum likelihood voxelwise weightings for each of the spectral components, we first note the posterior distribution for the model, up to proportionality

$$\pi(\theta|\mathbf{D}) \propto \pi(\mathbf{D}|\theta)\pi(\theta) = \prod_{n=1}^{N} \mathcal{N}\left(\mathbf{S}_n; \mathbf{K}F(\mathbf{z}_n), \sigma_n^2 I\right) \tag{30}$$

where we have assumed a uniform prior on all parameters θ. The posterior distribution for each $\mathbf{z}_n$ - up to proportionality - is therefore

$$\pi(\mathbf{z}_n|\mathbf{S}_n, \theta^{(t-1)}) \propto \mathcal{N}\left(\mathbf{S}_n; \mathbf{K}F(\mathbf{z}_n), \sigma_n^2 I\right) \tag{31}$$

We therefore update $\mathbf{z}_n = \{z_{nm}\}_{m=1}^{M}$ by maximising this (in log-scale), subject to $\sum_{m=1}^{M} z_{nm} = 1$, i.e.

$$\mathbf{z}_n = \underset{\sum_{m=1}^{M} z_{nm}=1}{\arg\max} \log \mathcal{N}\left(\mathbf{S}_n; \mathbf{K}F(\mathbf{z}_n), \sigma_n^2 I\right) \tag{32}$$

which we solve sequentially for voxels $n = 1, ..., N$ with the interior-point algorithm.

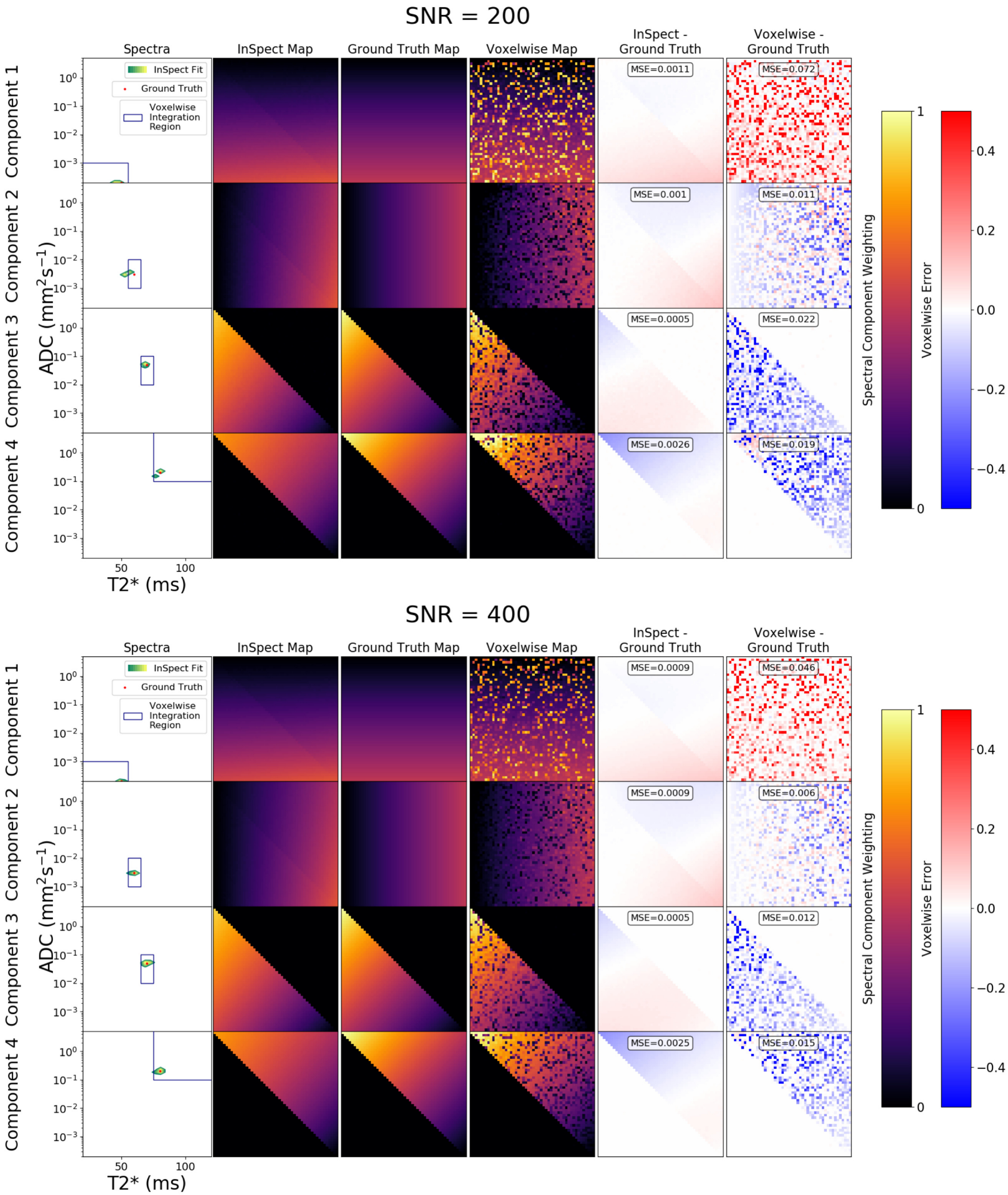

Fig. S1. As Figure 2 but for simulations with higher SNR.

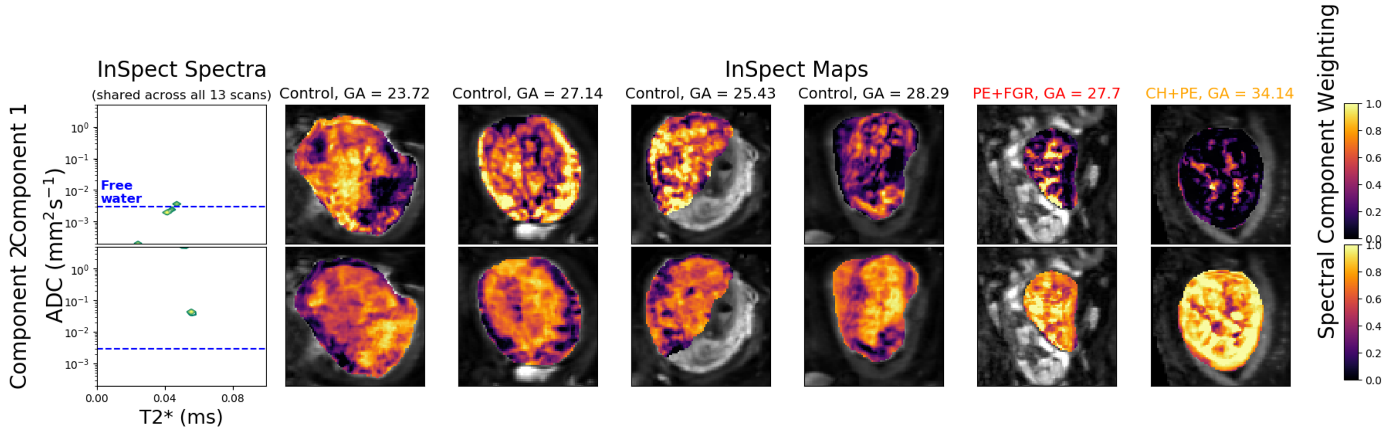

Fig. S2. As Figure 5 but for the two-component InSpect run on 13 placenta diffusion-relaxometry scans.

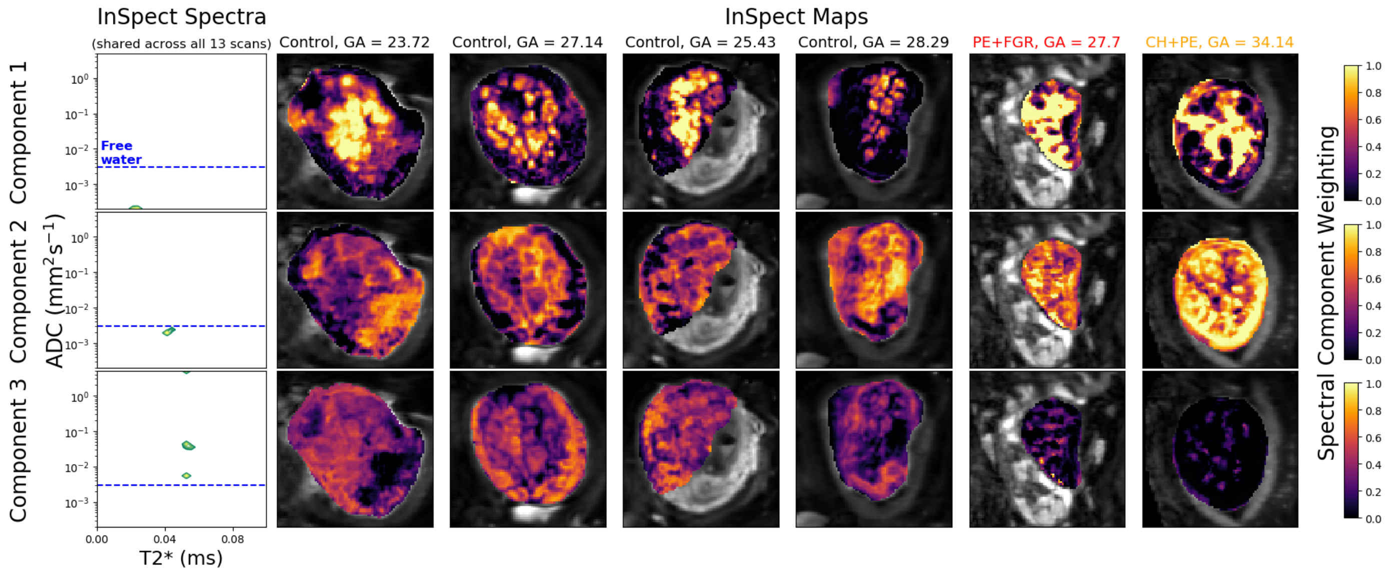

Fig. S3. As Figure 5 but for the three-component InSpect run on 13 placenta diffusion-relaxometry scans.

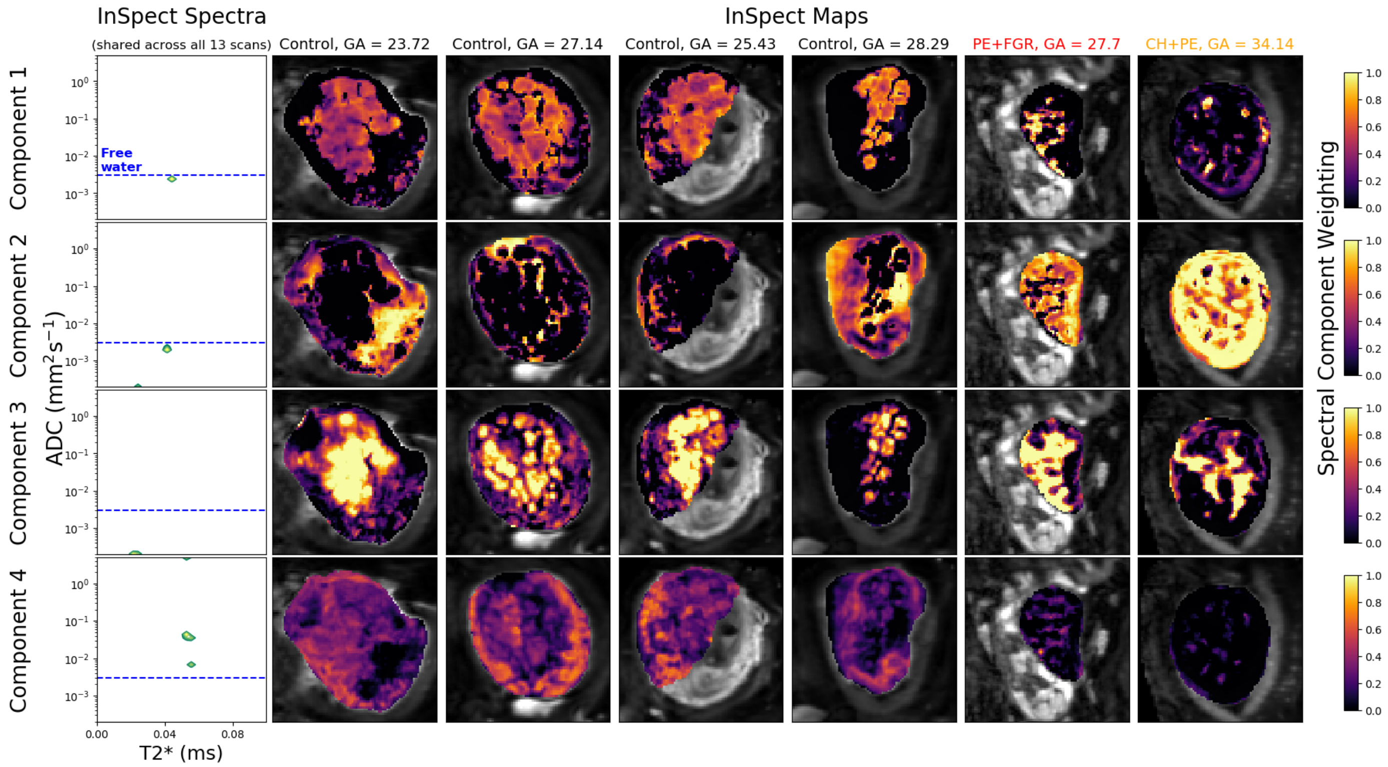

Fig. S4. As Figure 5 but for the four-component InSpect run on 13 placenta diffusion-relaxometry scans.

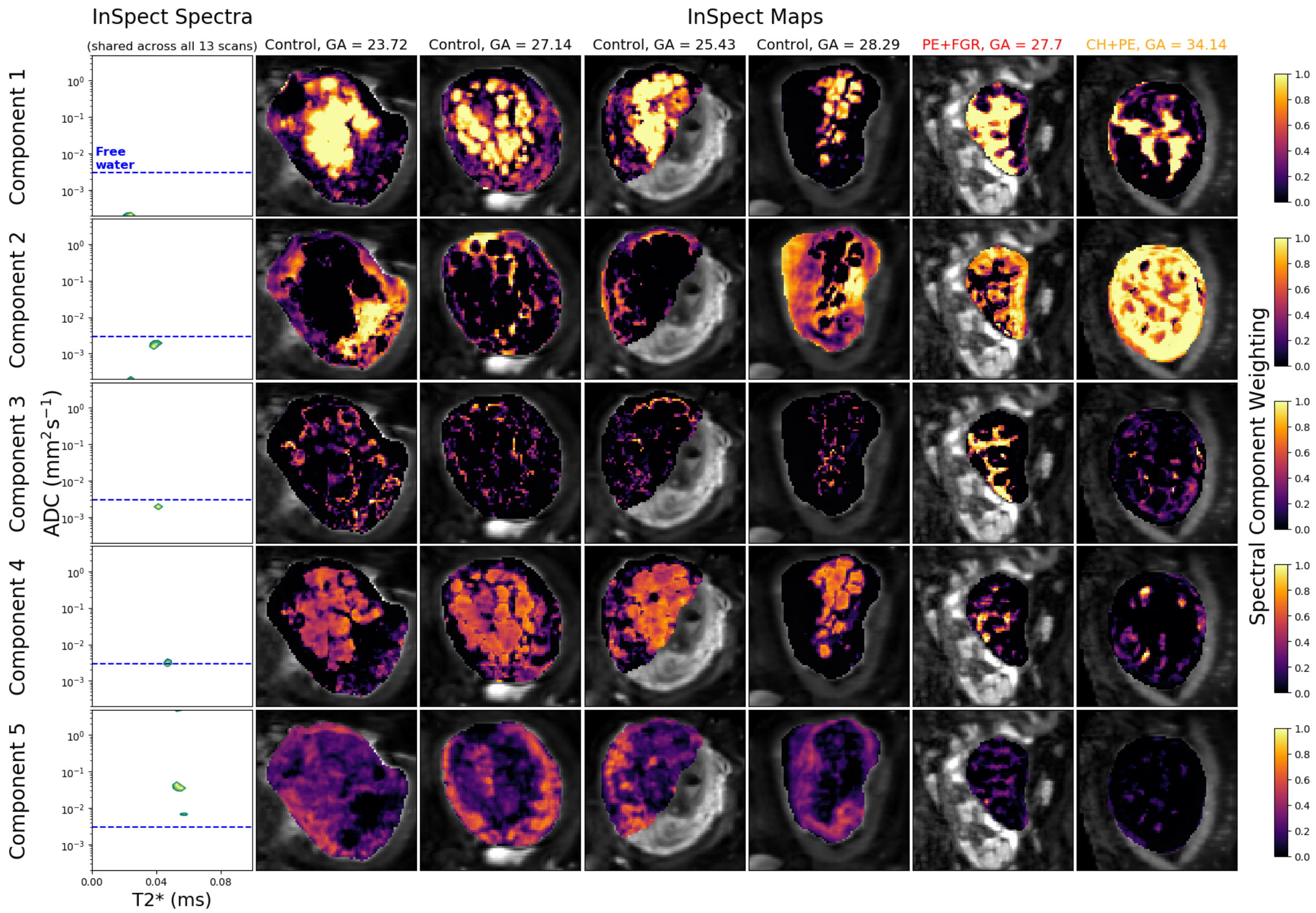

Fig. S5. As Figure 5 but for the five-component InSpect run on 13 placenta diffusion-relaxometry scans.

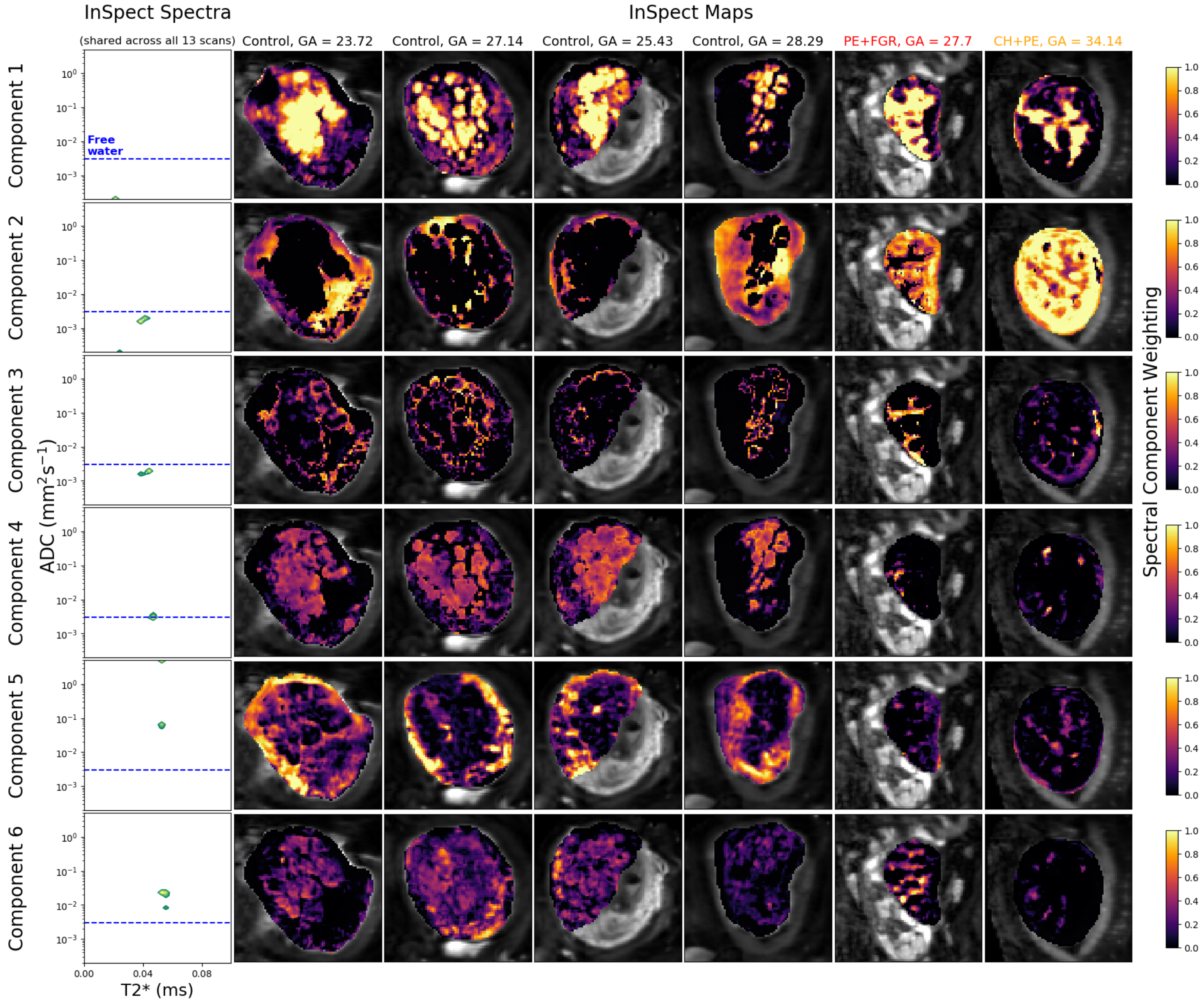

Fig. S6. As Figure 5 but for the six-component InSpect run on 13 placenta diffusion-relaxometry scans.

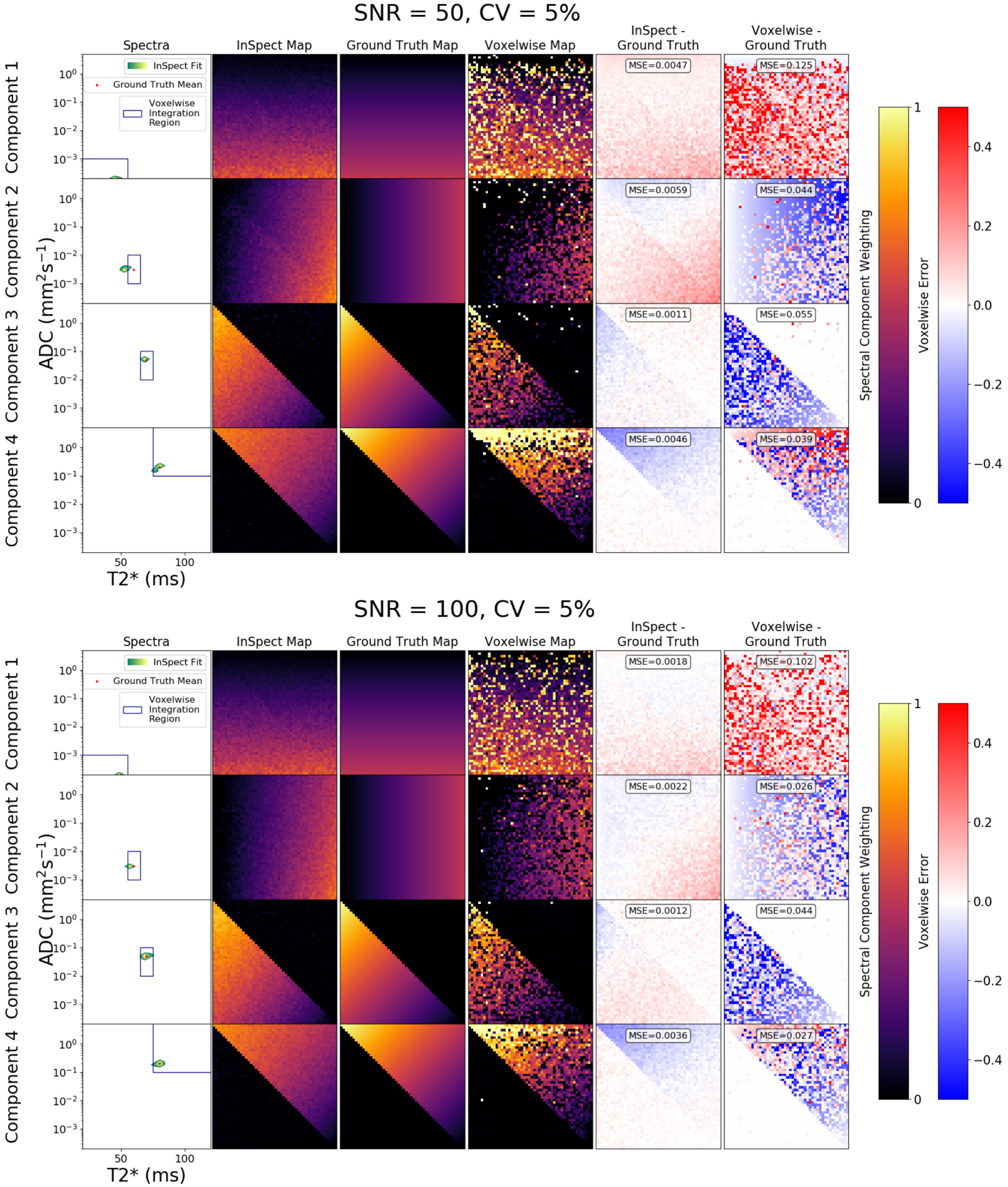

Fig. S7. As Figures 2 and S1, except with voxelwise noise added to the underlying component spectra (in addition to the image-level noise corresponding to the SNR value). In each voxel, normally-distributed noise with a 5% coefficient of variation was added to the ADC and T2* values for each component's spectra. See Figure S8 for corresponding results on simulations with higher SNR.

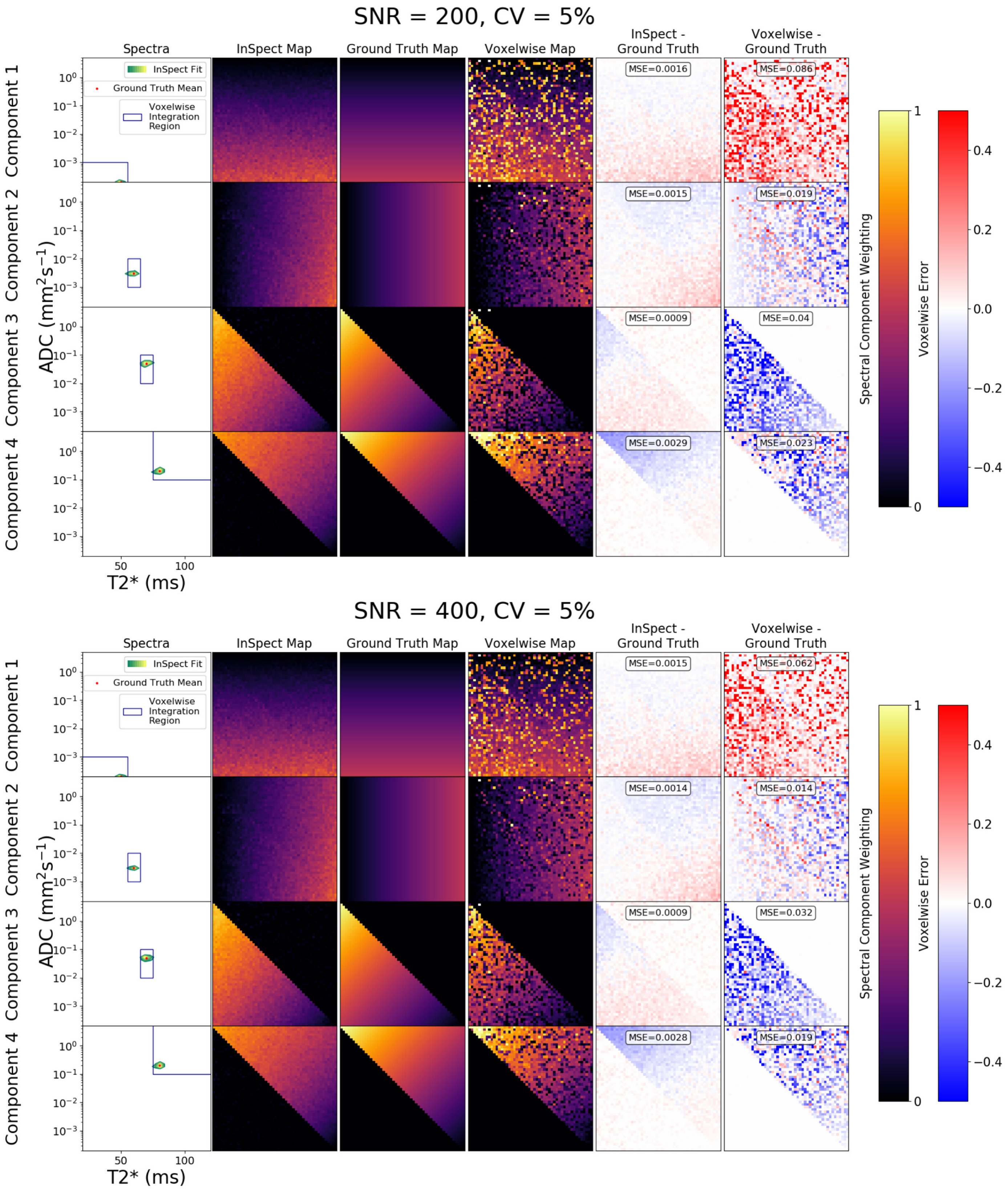

Fig. S8. As Figure S7 except with higher image SNR.
